# Dr. Oliver Wendell Holmes

**Published:** 1894-10

**Authors:** 


### DR. OLIVER WENDELL HOLMES.

Medicine has given to the world not a few men of literary genius, outside their chosen profession. Among them, none more gifted, none more loved than the late Dr. Oliver Wendell Holmes. That a physician should find time for literary work, even in the field of medicine, is a difficult task, being as he is ever at the beck and call of a capricious public, having no time either night or day that he can call his own. True, the latter years of Dr. Holmes's life were devoted almost exclusively to literary work, and thus his professional career has been overshadowed, and with the general public his reputation is as author and poet, and with them only the title of his profession is enduring.

It may not be uninteresting to the physician to glance briefly at the life of Dr. Holmes as a doctor. The son of a minister, he first studied law, but early abandoned his profession and graduated as M. D. in 1836; while he spent two years in Paris in the prosecution of his studies, he returned to America and received his diploma from Harvard. For forty-four years he was a teacher of medical students; occupying the chair of anatomy and physiology in his *alma mater* from 1847 to 1882, he was continuously engaged in the scientific study of medical subjects and was a frequent contributor to medical journals. Together with Professor Bigelow, he edited Marshall Hall's *Principles of the Theory and Practice of Medicine.* Among his scientific works may be mentioned, *Currents and Counter-Currents in Medical Science* (1861). *Lectures on Homeopathy and its Kindred Delusions* (1842). *Puerperal Fever as a Private Pestilence* (1855). *Border-Lines in Some Provinces of Medical Science* (1862), *etc.*

His writings are in plain and simple language, his power of presentation remarkably clear. He was a frequent lecturer before societies and college commencements. He was beloved of all who knew him, and though probably most widely known as the author of that most charming of books, *The Autocrat of the Breakfast Table,* yet he will live in the hearts of the profession and of his students as their scientist and physician.

